# Temporal and spectral characteristics of dynamic functional connectivity between resting-state networks reveal information beyond static connectivity

**DOI:** 10.1371/journal.pone.0190220

**Published:** 2018-01-10

**Authors:** Sharon Chiang, Emilian R. Vankov, Hsiang J. Yeh, Michele Guindani, Marina Vannucci, Zulfi Haneef, John M. Stern

**Affiliations:** 1 Department of Statistics, Rice University, Houston, Texas, United States of America; 2 Baylor College of Medicine, School of Medicine, Houston, Texas, United States of America; 3 Baker Institute for Public Policy, Rice University, Houston, Texas, United States of America; 4 Department of Neurology, University of California at Los Angeles, Los Angeles, California, United States of America; 5 Department of Statistics, Uniersity of California at Irvine, Irvine, California, United States of America; 6 Department of Neurology, Baylor College of Medicine, Houston, Texas, United States of America; 7 Neurology Care Line, Michael E. DeBakey VA Medical Center, Houston, Texas, United States of America; University of Texas at Austin, UNITED STATES

## Abstract

Estimation of functional connectivity (FC) has become an increasingly powerful tool for investigating healthy and abnormal brain function. Static connectivity, in particular, has played a large part in guiding conclusions from the majority of resting-state functional MRI studies. However, accumulating evidence points to the presence of temporal fluctuations in FC, leading to increasing interest in estimating FC as a dynamic quantity. One central issue that has arisen in this new view of connectivity is the dramatic increase in complexity caused by dynamic functional connectivity (dFC) estimation. To computationally handle this increased complexity, a limited set of dFC properties, primarily the mean and variance, have generally been considered. Additionally, it remains unclear how to integrate the increased information from dFC into pattern recognition techniques for subject-level prediction. In this study, we propose an approach to address these two issues based on a large number of previously unexplored temporal and spectral features of dynamic functional connectivity. A Generalized Autoregressive Conditional Heteroskedasticity (GARCH) model is used to estimate time-varying patterns of functional connectivity between resting-state networks. Time-frequency analysis is then performed on dFC estimates, and a large number of previously unexplored temporal and spectral features drawn from signal processing literature are extracted for dFC estimates. We apply the investigated features to two neurologic populations of interest, healthy controls and patients with temporal lobe epilepsy, and show that the proposed approach leads to substantial increases in predictive performance compared to both traditional estimates of static connectivity as well as current approaches to dFC. Variable importance is assessed and shows that there are several quantities that can be extracted from dFC signal which are more informative than the traditional mean or variance of dFC. This work illuminates many previously unexplored facets of the dynamic properties of functional connectivity between resting-state networks, and provides a platform for dynamic functional connectivity analysis that facilitates its usage as an investigative measure for healthy as well as abnormal brain function.

## Introduction

Functional connectivity (FC) between intrinsic functional networks is of increasing interest for understanding the human brain. Recent work suggests that these networks are identifiable during tasks as well as during the task-free resting state [1–3]. Furthermore, evidence has consistently found that connectivity between these networks at rest is informative, not only with respect to normal variations in cognitive function such as memory [4, 5], but also individual variations in abnormal and healthy brain function [6–10]. Several studies have indicated that the default mode network (DMN), which regulates introspective thought, is “anticorrelated” with other resting-state networks [2, 11]. The nature of these resting-state network interactions is thought to play a large role in facilitating normal cognitive function [5, 11].

Until recently, however, the majority of resting-state functional connectivity studies have been based on static measures of connectivity, which rely on the inherent assumption that inter-regional signal associations are constant over the length of the scan. With the discovery that inter-regional signal associations fluctuate over time both within and across scanning sessions [12, 13], increased attention has been paid towards understanding the dynamic properties of functional connectivity. Static measures of connectivity have been found to not fully reflect the temporal dynamics of connectivity [14], with a significant degree of temporal variability in the degree of anticorrelation between the DMN and other resting-state networks [15]. Further developments have found that the level of variability in dFC may itself be informative about brain function in normal aging [16], as well as disorders such as schizophrenia [13], major depressive disorder [17], and temporal lobe epilepsy [18]. However, current understanding of dFC is limited for several reasons. Firstly, the vast majority of analyses have utilized a sliding-window approach to estimate dynamic connectivity, which tends to produce artificial fluctuations in connectivity [19]. Recently, some efforts have been made to separate dynamic fluctuations caused by true changes versus those caused by statistical uncertainty [13, 18, 20]. Model-based approaches, such as Generalized Autoregressive Conditional Heteroskedasticity (GARCH) models, were investigated by [21] and found to provide more reliability than sliding-window approaches for dFC detection due to decreased sensitivity to parameter settings and susceptibility to noise. Secondly, the majority of studies have generally focused on a rather narrow set of characteristics of dFC temporal dynamics—typically the mean, variance, or number of state-to-state transitions in the time-domain. Recently, some studies have begun modeling dFC in the time-frequency domain using wavelet transform coherence [15, 22]. These studies suggest that additional information may be gained by considering spectral properties such as the number of time-frequency points spent in various clustered states. However, beyond this small set of features, much remains unknown about the temporal and spectral characteristics of dFC.

The aims of this study are to (1) investigate previously unexplored temporal and spectral characteristics of dynamic functional connectivity between the DMN and several other commonly investigated resting-state networks; (2) examine which temporal/spectral aspects of dynamic functional connectivity are altered in temporal lobe epilepsy; and (3) propose a new approach for integrating estimates of dFC into pattern recognition techniques for subject-level prediction, using as a feature vector the temporal and spectral features of dFC. GARCH is first used to estimate dFC between the DMN and other resting-state networks. Next, dFC is transformed into the spectral domain using Welch’s power spectral density estimate. Temporal and spectral features drawn from signal processing literature are then computed for the estimated dFC and corresponding spectral density. Lastly, Random Forests is used to assess the predictive accuracy of the proposed method for identifying disease states. Our proposed approach of investigating dFC in terms of its temporal and spectral features elucidates many previously unexplored facets of the normal dynamics of functional connectivity in fMRI. Furthermore, we show that the proposed approach provides an interpretable method for incorporating the new field of dFC into clinical outcome prediction on the individual subject level, which achieves superior performance compared to traditional estimates of the dFC variance or static connectivity. We illustrate our approach using connectivity between the default mode network and several commonly investigated resting-state networks in temporal lobe epilepsy.

## Materials and methods

### Participants and ethics statement

Participants consisted of 23 healthy controls (HC; average age, 31.1±6.5 SE (y); age range, 19-44 (y); 8 females) and 25 patients with temporal lobe epilepsy (TLE; average age, 33.6±7.8 SE (y); age range, 20-45 (y); 12 females; average epilepsy duration, 18.74±2.4 SE (y); epilepsy duration range, 2-39 (y)). Healthy control subjects had normal structural MRIs, no history of neurologic illness, and were not taking neurologic medications. All subjects were right-handed except four TLE patients. TLE patients were recruited from the University of California, Los Angeles (UCLA) Seizure Disorder Center. Diagnostic evaluation for all patients included video-EEG monitoring, high-resolution MRI, FDG-PET scanning, and neuropsychological testing. Written informed consent was obtained prior to scanning for all subjects in accordance with guidelines from the UCLA Institutional Review Board. The study protocol and consent procedure was approved by the UCLA Institutional Review Board (IRB) #10-000568. A two-sample *t*-test with unequal variances and Fisher exact test showed no significant difference in age, gender, or handedness at the *α* = 0.05 level of significance.

### Image acquisition and pre-processing

Imaging was performed with a 3T MRI system (Siemens Trio, Erlangen, Germany). Functional imaging was performed with the following parameters: TR = 2000 ms, TE = 30 ms, FOV = 210 mm, matrix = 64 × 64, slice thickness 4 mm, 34 slices. Subjects were instructed to relax with eyes closed during imaging, with concomitant EEG monitoring to confirm awake status. No auditory stimulus was present except for the acoustic noise from imaging. High-resolution structural images were obtained during the same imaging study with the parameters: TR = 20 ms, TE = 3 ms, FOV = 256 mm, matrix = 256 × 256, slice thickness 1 mm, 160 slices. The images were acquired in the axial plane using a spoiled gradient recalled (SPGR) sequence for the anatomical images and an echo planar imaging (EPI) sequence for the functional images. The imaging sessions included multiple simultaneous EEG and fMRI recordings, each lasting 5 to 15 minutes. The session with the least amount of motion was selected to include 10 to 15 minutes of BOLD fMRI data for each subject. To limit the influences of motion, image results were checked to ensure that no subjects had a maximum translation of > 1.5mm (HC, 0.24 ± 0.04 mm; TLE, 0.37 ± 0.04 mm). Patients remained on their regular medications during the fMRI. None of the patients had a seizure in the 24 hours preceding the imaging and none had seizures during the study as confirmed by the simultaneous EEG obtained during fMRI. The EEG results were not included in the data analysis other than to exclude seizures. Details of the simultaneous EEG methods have been described previously [23].

Neuroimaging and fMRI pre-processing steps are similar to that described previously [24]. Preprocessing was performed using FSL (fMRIB Software Library) version 5.0.7 (Oxford, United Kingdom, www.fmrib.ox.ac.uk/fsl) [25, 26] and included head movement artifact correction [27], non–brain tissue elimination [28], high-pass filtering (100 s), spatial smoothing at 5 mm full-width half-maximum, and mean-based intensity normalization as described previously for resting-state fMRI analyses [2, 11]. Excessive head movement was corrected using motion scrubbing through nuisance regression. TRs that showed instantaneous changes in blood oxygen level-dependent (BOLD) intensity that exceeded threshold (75th percentile + 1.5× interquartile range) were identified using FSL and added as single-timepoint nuisance regressors for motion censoring [29]. The average number of identified outliers per participant was 4.11% ± 2.65%. Tissue-type segmentation was performed on each participant’s structural image using FAST (FMRIB’s Automated Segmentation Tool) [30], before being aligned to their respective BOLD images. The first eigenvectors of the white matter signal and cerebrospinal fluid signal were obtained using the segmented masks. The following were included as temporal covariates and regressed out using linear regression: motion outliers, six motion parameters, white matter eigenvectors, cerebrospinal fluid eigenvectors, and their associated derivatives. Recent work suggests that, while resting-state networks are dominated by low-frequency fluctuations, the spectral range of resting-state networks contains meaningful information in spectral components up to and possibly beyond 0.25 Hz [31–33]. In the interest of capturing meaningful higher frequency information, a temporal low-pass filter was not applied to the data [34]. Residuals were used in the seed-based correlation analysis below.

### Extraction of resting-state networks

**Fig 1:**
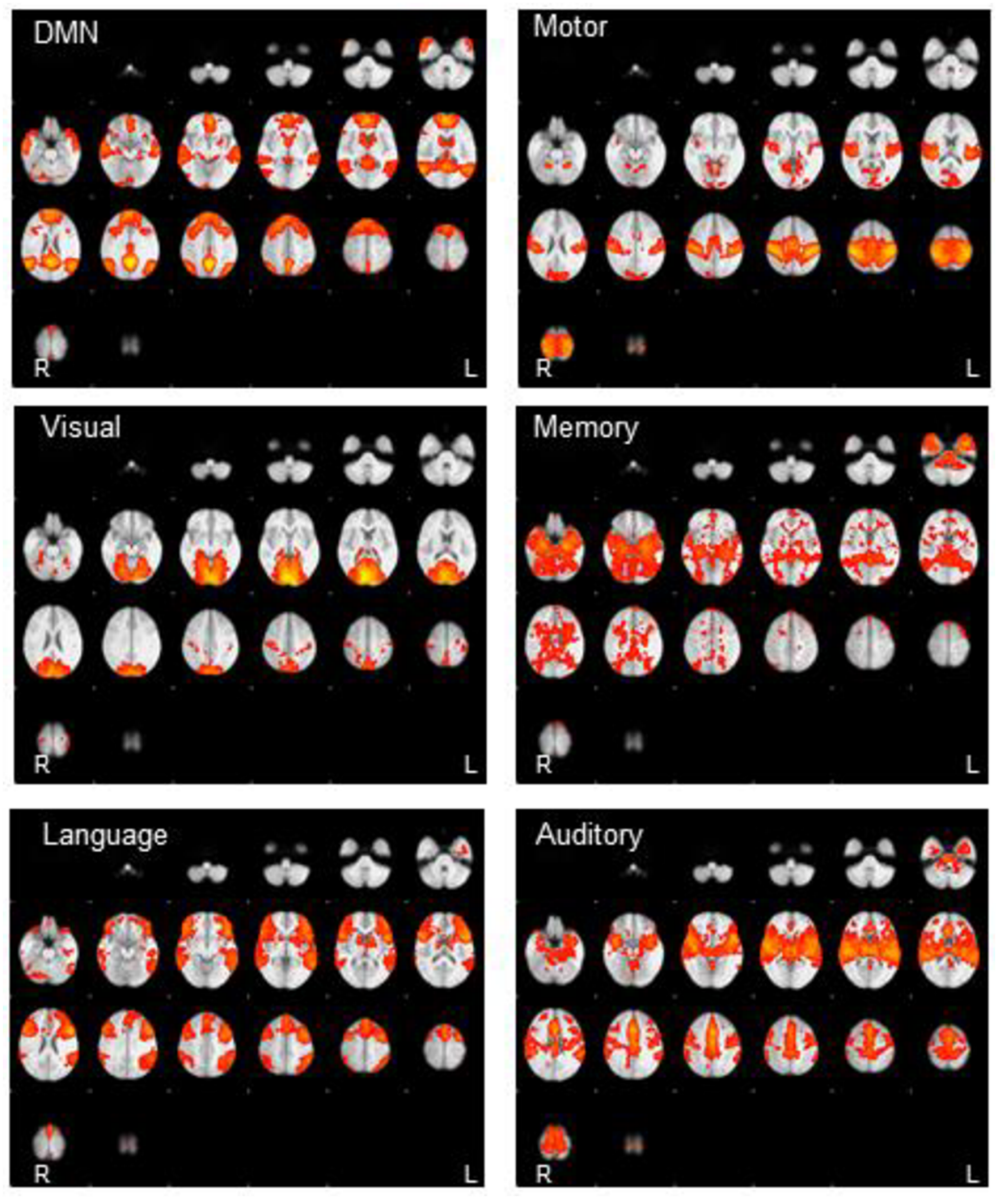
Resting-state connectivity patterns of resting-state networks across the whole group of subjects, overlaid in MNI space. Network seed coordinates and descriptions are provided in Table 1. Images are displayed in radiologic convention.

Functional connectivity networks were extracted for several commonly studied resting-state networks using a seed-based analysis, including the motor, visual, memory, language, auditory, and default mode networks. Seed-based correlation and spatial independent component analysis (ICA) are two predominant methods used for extracting networks from fMRI data. While seed-based and ICA approaches have distinct strengths, they have been found to yield similar networks for resting-state networks such as the motor, visual, default mode, and attention networks [35]. Seeds related to each network were defined *a priori* based on the literature as 6-mm spheres in MNI space (Table 1). For selected ROIs, bilateral seeds were created by mirroring the contralateral side of the seed and were subsequently merged. We also explored spheres with radii of 4-mm and 8-mm, and verified that the extracted networks were robust to radius size. These seeds were transformed from standard MNI space to each subject’s individual BOLD space. The mean time course of each seed was computed by averaging across all voxels within the seed and used as the dependent variable in a General Linear Model (GLM) as implemented by FSL’s FEAT, to obtain *Z*-statistic images. *Z*- (Gaussianized *T* or *F*) statistic images were thresholded using clusters determined by *Z* > 2.0 and a (corrected) cluster significance threshold of *p* < 0.05. Positively correlated voxels were used to construct network maps [2, 36, 37], which provided the final ROIs for dynamic connectivity analysis. Due to spatial overlap between networks, a winner-take-all approach was used in which overlapping voxels were assigned to the network with the greater correlation. The BOLD time-series within each session-wise network *Z*-map was extracted and averaged over all voxels in the session-wise network *Z*-map using a weighted average, with weights proportional to the degree of correlation with the seed voxel. The weighted approach allows for greater spatial homogeneity compared to nonweighted networks, while relaxing the constraint for spatial contiguity enforced by using the original spherical seeds as ROIs. Additionally, group analysis was performed to characterize the functional networks across all subjects (Fig 1).

**Table 1:** Montreal Neurological Institute (MNI) coordinates and abbreviations of resting-state networks.

Network	Abbreviation	Seed
Visual	VIS	Calcarine fissure: (-9,-89,1),(12,-88,1)
Default mode	DMN	Posterior cingulate cortex [69]: (2,-60,36)
Language	LANG	Pars opercularis [14, 70]: (-49, 13, 18)
Auditory	AUD	Primary auditory cortex [14, 71]: (-41,-22,6), (43,-22,6)
Motor	MOT	Primary motor cortex [14, 72]: (-42,-28,53),(43,-28,53)
Memory	MEM	Hippocampus: (-23,-15,-23), (26,-15,-23)

**Fig 2:**
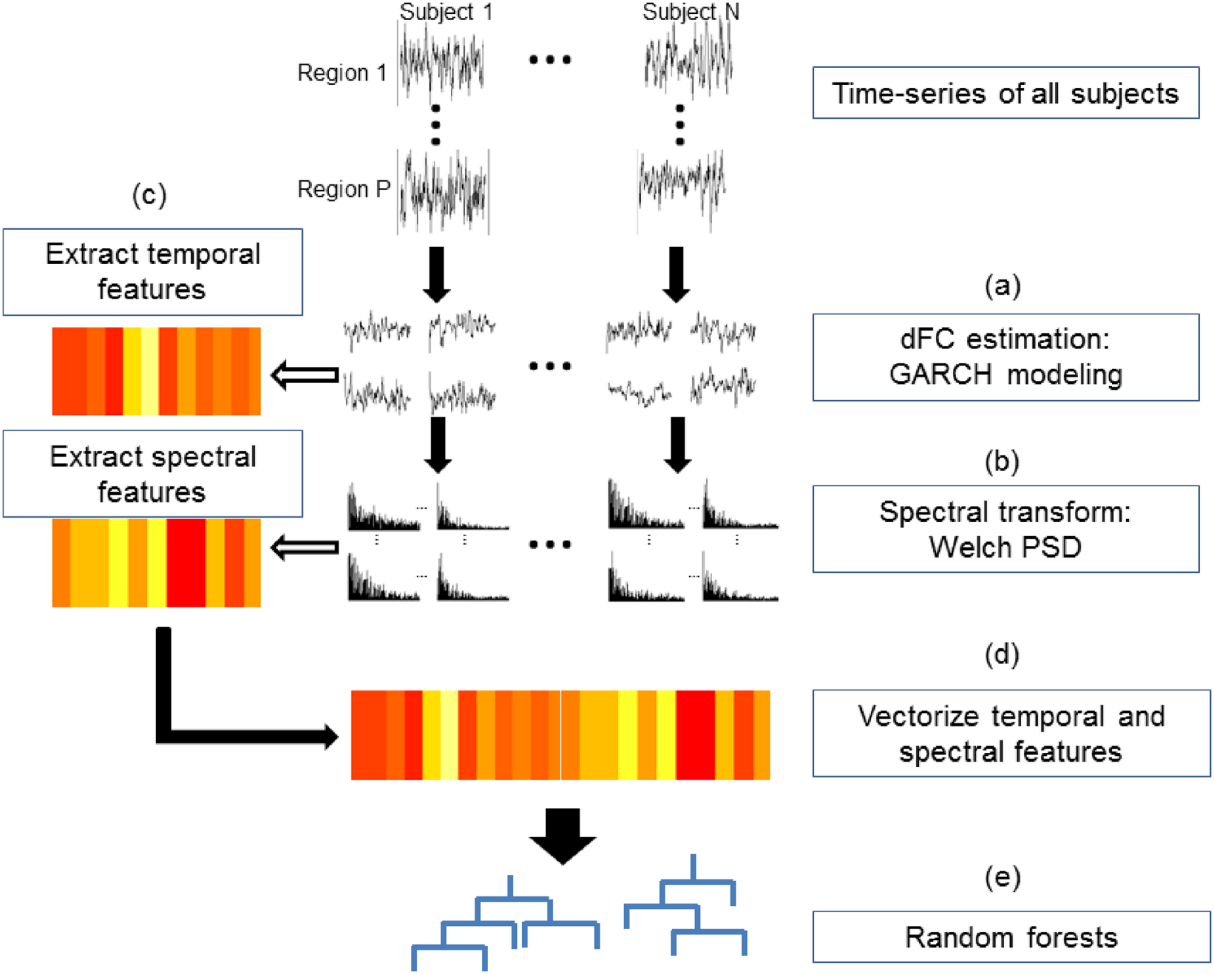
Schematic of proposed framework for studying temporal and spectral characteristics of dynamic functional connectivity. (a) Dynamic functional connectivity (dFC) is first estimated from a model-based state-space approach. (b) dFC estimates are transformed to the spectral domain using Welch’s power spectral density estimate. (c) Temporal and spectral features are extracted from the temporal and spectral domains. (d) Temporal and spectral features are concatenated and (e) used as the feature vector for subject-level prediction.

A visual overview of the methods can be found in Fig 2. Estimates of dynamic functional connectivity are first obtained using a model-based approach to estimate dFC (Fig 2a). Although a GARCH model is employed here, other popular dFC estimation approaches, including sliding window techniques or other model-based approaches, could be used at this step. Next, the estimated dFC is transformed into the frequency domain using Welch’s method for spectral density estimation (Fig 2b). We extract temporal and spectral features of dFC from the resulting signal (Fig 2c). These temporal and spectral features of dFC are then concatenated and used within pattern recognition techniques (Fig 2d and 2e). In the following sections, we describe each of these steps in more detail.

### Dynamic Conditional Correlation Model

To model dynamic functional connectivity between resting-state networks, we consider the Dynamic Conditional Correlation (DCC) model of [38]. Let ***Z***_*it*_ = (*Z*_1*it*_, *Z*_2*it*_)′ be a random vector representing a pair of BOLD time series of any two ROIs in the brain at time *t*, for each of *i* = 1, …, *N* subjects. For simplicity, below we omit the index *i*. Although the methods described in the following sections may be applied to any choice of networks or regions, in this study we investigate dynamic functional connectivity between the default mode network (DMN) and the commonly investigated resting-state networks shown in Table 1, including the visual, language, auditory, motor, and memory networks.

We model the dynamic relationship between any pair of networks with the following process:

$$\begin{array}{ccc} Z_{t} & = & \mu_{t}+\epsilon_{t}\;\;\;\;\;\;\;\;1\leq t\leq T. \end{array}$$ (1)

In our work we assume that an ARMA(2,2) mean process is consistent with our data. In particular we let each element of the mean vector *μ*_*t*_ to be defined as

$$\begin{array}{ccc} \mu_{jt} & = & \alpha_{1}+\beta_{1}Z_{j(t-1)}+\beta_{2}Z_{j(t-2)}-\theta_{1}\epsilon_{j(t-1)}-\theta_{2}\epsilon_{j(t-2)}\;\;\text{for}\;\;j=1,2. \end{array}$$

Ljung-Box test and autocorrelation residual analysis were performed and confirmed our assumption regarding the mean process of the time series. Further, we let the covariance matrix associated with ***ϵ***_*t*_ for each subject to be given by:

$$\begin{array}{c} \Sigma_{t}=D_{t}R_{t}D_{t}, \end{array}$$ (2)

where ***D***_*t*_ = diag(*σ*_1*t*_, *σ*_2*t*_).

The correlation matrix ***R***_*t*_ contains the correlation coefficient *ρ*_*t*_ representing the dynamic connectivity between two regions *Z*_1*t*_ and *Z*_2*t*_ for every subject *i*. For the conditional covariance we assume a GARCH-DCC process of order one:

$$\begin{array}{c} \sigma_{jt}^{2}=\omega_{j}+\phi_{j}Z_{jt}^{2}+\psi_{j}\sigma_{j(t-1)}^{2},\;\;\;\text{for}\;\;j=1,2. \end{array}$$ (3)

To complete the DCC specification of the model we further let:

$$u_{t}=D_{t}^{-1}\epsilon_{t}$$ (4)

$$Q_{t}=\left(1-\eta_{1}-\eta_{2}\right)\Xi +\eta_{1}u_{t}u_{t}^{\prime }+\eta_{2}Q_{t-1}$$ (5)

$$R_{t}=\text{diag}{\left(Q_{t}\right)}^{-1/2}Q_{t}\text{diag}{\left(Q_{t}\right)}^{-1/2},$$ (6)

where Ξ represents the unconditional correlation matrix of *u*_*t*_ and 0 < *η*_1_ + *η*_2_ < 1.

The estimation of the model parameters ***λ*** = (*α*_1_, *α*_2_, *β*_1⋅_, *β*_2⋅_, *θ*_1⋅_, *θ*_2⋅_, *ω*_1_, *ω*_2_, *ϕ*_1_, *ϕ*_2_, *ψ*_1_, *ψ*_2_) and the dynamic conditional correlations ***R***_*t*_ are of interest. The likelihood function of the model can be written as:

$$\begin{array}{ccc} L(Z_{t}|\lambda ) & = & -\frac{1}{2}\sum_{t=1}^{T}\left(nlog\left(2\pi \right)+log\right|\Sigma_{t}\left|+Z_{t}^{\prime }\Sigma_{t}Z_{t}\right)\\ & = & -\frac{1}{2}\sum_{t=1}^{T}\left(nlog\left(2\pi \right)+log\right|D_{t}{|}^{2}+Z_{t}^{\prime }D_{t}^{-2}Z_{t}-u_{t}^{\prime }u_{t}+log|R_{t}\left|+u_{t}^{\prime }R_{t}^{-1}u_{t}\right). \end{array}$$ (7)

The first three terms above involve only the variance and the last three only the correlation. Moreover, the variance terms of the likelihood can be estimated using a univariate GARCH model for each *Z*_*jt*_, *j* = 1, 2. This motivates the use of a two-step procedure for the estimation of all parameters of the DCC-GARCH mode as discussed in [38]. In the first step, a univariate GARCH model is fit to the two time series individually. The residuals are then used to estimate the parameters entering the correlation terms in Eq (7).

### Feature extraction

The majority of fMRI functional connectivity studies are currently based on static functional connectivity or the mean or variance of dynamic functional connectivity. Dynamic functional connectivity contains a large amount of information which may be of use in increasing the amount of signal extracted from fMRI connectivity analysis. However, direct usage of dynamic functional connectivity time-series estimates may not work well in machine learning for several reasons, including poor interpretability and noisiness of the raw time-series. *Feature engineering* provides a commonly employed approach in signal processing literature, in which domain knowledge is used to transform an otherwise noisy raw signal into simpler attributes with higher predictive power.

Here, we use feature engineering to evaluate whether additional informative features can be extracted from dFC signal in addition to the traditionally employed mean and variance. To do so, we extract a large number of temporal and spectral features drawn from acoustic and EEG signal processing literature. Due to the usefulness of these features in acoustic and EEG analysis [39–41], we hypothesized that these features may also be useful for extracting information from dynamic functional connectivity.

Table 2 summarizes the dynamic functional connectivity features investigated. The computation of each feature is described in detail below (see *Temporal dFC features* and *Spectral dFC features* sections). As dynamic funtional connectivity is a still emerging area of research, the physiologic interpretation of many of these features is not yet known; however, motivating physiologic interpretations are also discussed below when known.

**Table 2:** Temporal and spectral features of dynamic functional connectivity. Features and abbreviations are shown. dFC, dynamic functional connectivity.

Abbreviation	Feature	Interpretation
ALFF-dFC	Amplitude of Low Frequency Fluctuations in dFC	Energy of dFC power spectrum in low-frequency range (0.01-0.08 Hz)
CREST	Spectral Crest	Peakiness of dFC power spectrum
FLAT	Spectral Flatness	Noisiness of dFC power spectrum
FLUX	Spectral Flux	Rate of change of dFC power spectrum
KURT	Spectral Kurtosis	Kurtosis of dFC power spectrum
MV	Mean Value	Average magnitude of dFC
PAV	Proportion of Anticorrelated Volumes	Proportion of time points for which dFC is negative
PEAK	Dominant Frequency	Most prominent frequency in dFC
PSE	Power Spectral Entropy	Entropy of dFC power spectrum
SCO	Spectral Centroid	Center of mass of dFC power spectrum
SLOPE	Spectral Slope	Rate of change of dFC power spectrum toward higher frequencies
SMED	Median Frequency	Median frequency of dFC power spectrum
SKW	Spectral Skewness	Skewness of dFC power spectrum
SPR	Spectral Spread	Measure of bandwidth of dFC power spectrum
SRO	Spectral Rolloff	Right-skewness of dFC power spectrum
VAR	Variance	Variance of dFC fluctuations
ZC	Zero-Crossing Rate	Rate at which dFC changes signs

All code was written in R version 3.1.3. R code to carry out implementation is available at the corresponding author’s website.

### Temporal dFC features

Static connectivity measures the average value of the statistical dependence between two time-series over a given time period [42]. To capture this, the *mean value* (MV) of the estimated dynamic functional connectivity was computed for every subject, defined as the average magnitude of the dynamic functional connectivity. Based on recent observations that the variance in dFC may also be informative about brain function [13, 17, 18], we also compute a *variance* (VAR) feature which is calculated as the unbiased sample variance of dFC:

$$\begin{array}{c} {\text{MV}}_{i}=\frac{1}{T}\sum_{t=1}^{T}R_{it} \end{array}$$ (8)

$$\begin{array}{c} {\text{VAR}}_{i}=\frac{1}{T-1}\sum_{t=1}^{T}{\left(R_{it}-MV_{i}\right)}^{2}. \end{array}$$ (9)

The mean value and variance of dFC have typically been considered in dFC research. To examine whether additional information is present in dFC signal that can improve prediction, we examine additional temporal and spectral quantities previously unexplored in dFC research. Two additional time-domain features of dFC were considered: (a) *proportion of anticorrelated volumes* (PAV), which is defined as the proportion of time points for which dFC is negative in sign, capturing the degree of “anticorrelation” between networks, and (b) *zero-crossing rate* (ZC), which provides a measure of the rate at which dFC changes signs (e.g. from positive to negative and vice versa). The mathematical definitions for PAV and ZC for the *i*th subject are given in equations Eqs (10) and (11), where **1**_*R*_*it*_<0_ is an indicator function equal to 1 if *R*_*it*_ < 0, and 0 otherwise:

$$\begin{array}{c} {\text{PAV}}_{i}=\frac{1}{T}\sum_{t=1}^{T}1_{R_{it}<0} \end{array}$$ (10)

$$\begin{array}{c} {\text{ZC}}_{i}=\frac{1}{T-1}\sum_{t=1}^{T-1}1_{R_{it}R_{i,t-1}<0\cap \left|R_{it}-R_{i,t-1}\right|\geq c}, \end{array}$$ (11)

where *R*_*it*_ is the estimated dynamic functional connectivity for subject *i* at time point *t*, as described above (see *Dynamic Conditional Correlation Model*), and the threshold *c* was included in Eq (11) in order to avoid signal crossings due to low-level noise. Here, we set *c* = 0.001.

### Spectral dFC features

Welch’s method [43] was used to obtain power spectra of the estimated dynamic functional connectivity. We investigate the utility of several spectral features for dFC analysis. To capture the distributional properties of the power spectra, we examined the first four spectral central moments of dFC, including the (a) *spectral centroid* (SCO), which measures the center of mass of the dFC spectrum, with higher values indicating greater energy concentrated at higher frequencies; (b) *spectral spread* (SPR), which measures the bandwidth of the dFC spectrum; (c) *spectral skewness* (SKW), which measures the symmetry of the power spectral density, with positive (negative) values indicating positive (negative) skewness; and (d) *spectral kurtosis* (KURT), which measures the distribution of frequencies around the spectral centroid, with higher values indicating dFC frequencies more highly clustered around the spectral centroid. Definitions are provided in Eqs (12)–(15):

$${\text{SCO}}_{i}=\frac{\sum_{k}f_{ik}M_{ik}}{\sum_{k}M_{ik}}$$ (12)

$${\text{SPR}}_{i}=\frac{\sum_{k}{\left(f_{ik}-SCO_{i}\right)}^{2}M_{ik}}{\sum_{k}M_{ik}}$$ (13)

$${\text{SKW}}_{i}=\frac{\frac{1}{\sum_{k}M_{ik}}\sum_{k}{\left(f_{ik}-SCO_{i}\right)}^{3}M_{ik}}{SPR_{i}^{1.5}}$$ (14)

$${\text{KURT}}_{i}=\frac{\frac{1}{\sum_{k}M_{ik}}\sum_{k}{\left(f_{ik}-SCO_{i}\right)}^{4}M_{ik}}{SPR_{i}^{2}}-3.$$ (15)

Here, *M*_*ik*_ and *f*_*ik*_ are the magnitude of the spectra at frequency bin *k* and the frequency of bin *k*, respectively, for subjects *i* = 1, …, *N*. In addition, we examined the *median frequency* (SMED), or the frequency at which the power spectrum is divided into two regions with equal amplitude. The median frequency and the spectral centroid are both measures of the central tendency of the spectral distribution. However, estimated values of SMED are less affected by random noise. The definition of SMED is given in Eq (16):

$$\begin{array}{c} \underset{{\text{SMED}}_{i}}{\text{argmin}}\sum_{k=1}^{{\text{SMED}}_{i}}M_{ik}\geq 0.5\sum_{k}M_{ik}. \end{array}$$ (16)

The *spectral rolloff* (SRO), a measure of the right-skewness of the power spectra, was also computed. SRO is the frequency below which 85% of the spectral distribution magnitude is concentrated, as given in Eq (17):

$$\begin{array}{c} \underset{{\text{SRO}}_{i}}{\text{argmin}}\sum_{k=1}^{{\text{SRO}}_{i}}M_{ik}\geq 0.85\sum_{k}M_{ik}, \end{array}$$ (17)

where SRO_*i*_ is the rolloff frequency.

Seven other features in the frequency domain were considered: (a) *dominant frequency* (PEAK), which is the dominant frequency of dFC oscillations, calculated as the peak with the largest average power in its bin [39]; (b) *spectral crest* (CREST), which measures the peakiness of the power spectral density; (c) *spectral flux* (FLUX), which measures the rate of change of the power spectrum; (d) *spectral slope* (SLOPE), which is the tendency for the signal to have less energy at higher frequencies, calculated through linear regression of Welch’s estimate for the magnitude spectrum on the frequencies; (e) *spectral flatness* (FLAT), which is a measure of the noisiness of the dFC magnitude spectrum and is calculated as the ratio between the geometric and arithmetic means; (f) *power spectral entropy* (PSE), which is calculated as the information entropy of the dFC power spectrum, with small values of PSE indicating a narrow spectral peak and large values indicating a more smooth spectral peak; and (g) *amplitude of low-frequency fluctuations in dFC* (ALFF-dFC), which is a measure of the energy of the dFC in the low-frequency range. ALFF-dFC is a derivation of the amplitude of low-frequency fluctuations (ALFF) [44], which was originally proposed as an index for fluctuations in BOLD intensity in given regions of the brain. Similar to ALFF, we computed ALFF-dFC as the square root of the power spectrum of dFC, averaged across the low frequency range of 0.01–0.08 Hz. The mean of the derived *R*_*t*_ was removed prior to estimating the power spectrum for features for which a peak at the zero frequency was not informative about the feature of interest (PEAK, ALFF-dFC).

### Temporal and spectral features of dFC in healthy controls and temporal lobe epilepsy patients

We investigated differences in each of the above dynamic functional connectivity features between TLE and healthy controls. S1 Table provides a simulation study to characterize the ability of the features to capture the true dynamic connectivity. Further details can be found in the supplementary material.

To compare each dynamic functional connectivity feature between healthy controls and TLE, confidence intervals for the difference in means were estimated through bias-corrected and accelerated (BCa) bootstrap confidence intervals. The BCa procedure is a nonparametric approach for estimating confidence limits from a bootstrap distribution, which is used to estimate confidence intervals in data in which the underlying distribution is unknown. The BCa approach improves coverage accuracy over the standard percentile interval approach for bootstrap confidence intervals by accounting for two additional parameters: a bias-correction factor, which estimates the median bias of statistic, and an acceleration factor which estimates the rate of change of the standard error of the statistic with respect to the true parameter [45]. The bootstrap distribution of the sample difference in means for each feature was drawn through the ordinary nonparametric bootstrap with 1000 resamples. Significant differences were identified through BCa bootstrap confidence intervals, with false discovery rate (FDR) control at the 0.05 level to correct for multiple comparisons [46]. Corresponding corrected *p*-values are also provided.

### Dynamic connectivity and class prediction

Next, we investigate the utility of the proposed features for improving disease prediction using dynamic functional connectivity. Temporal and spectral features are highly collinear, rendering prediction using traditional regression methods ill-posed. Therefore, we used the classifier Random Forests (RF) [47] to perform simultaneous variable selection and subject-level prediction. We assume a two-class classification problem (TLE versus healthy controls), with temporal and spectral features of the estimated dynamic functional connectivity as predictor variables *X*_*ij*_, where *i* = 1, …, *N* indexes subjects and *j* = 1, …, *p* indexes the temporal and spectral dFC features described above. Let *y*_*i*_ denote the disease class of subject *i*. The Random Forest algorithm proceeds by generating a sequence of weak learners, such as decision trees, on bootstrapped samples of the data. Each tree is built based on a random subset of the features. More specifically, if we let *G*_*m*_(*X*), for *m* = 1,…,*M* trees, be a sequence of learners, then the final prediction of the Random Forest algorithm is obtained by combining all learners via:

$$\begin{array}{c} G\left(x\right)=\text{majority}\;\text{vote}{\left\{G_{m}\left(x\right)\right\}}_{m=1}^{M}. \end{array}$$

Five-fold cross-validation was used to assess the predictive performance of all temporal and spectral features. Random Forest parameters were optimized within each training set using a two-dimensional grid search to minimize cross-validated error.

**Fig 3:**
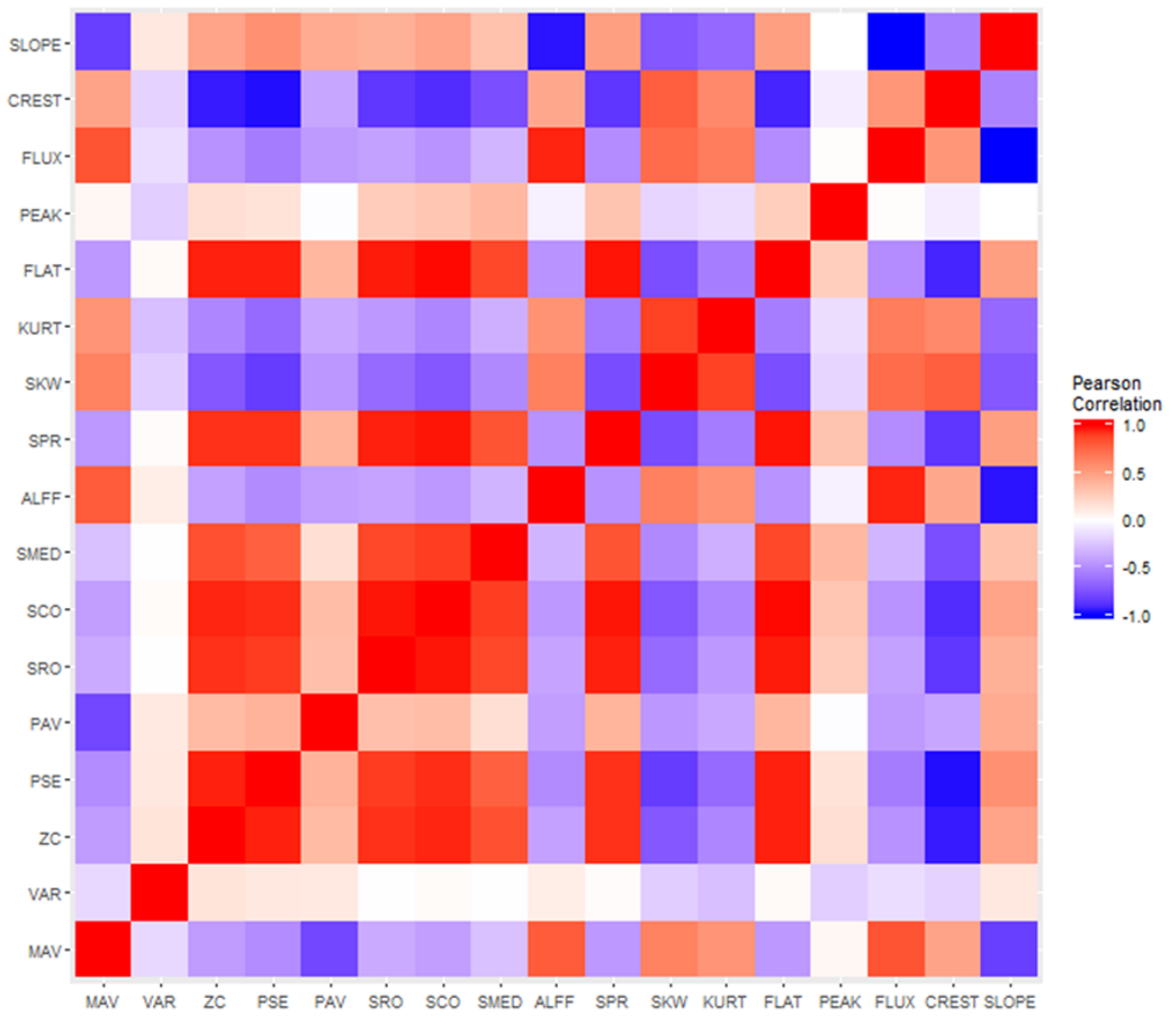
Pearson correlation heatmap of dFC features for DMN-memory network connectivity. Red indicates positive correlations; blue indicates negative correlations. *Abbreviations:* MV, Mean Value; VAR, Variance; ZC, Zero Crossing Rate; PSE, Power Spectral Entropy; PAV, Proportion of Anticorrelated Volumes; SRO, Spectral Rolloff; SCO, Spectral Centroid; SMED, Median Frequency; ALFF-dFC, Amplitude of Low Frequency Fluctuations in dFC; SPR, Spectral Spread; SKW, Spectral Skewness; KURT, Spectral Kurtosis; FLAT, Spectral Flatness; PEAK, Spectral Peak; FLUX, Spectral Flux; CREST, Spectral Crest; SLOPE, Spectral Slope.

To assess the relative contribution of the proposed dFC features for differentiating TLE from healthy controls, relative importance was quantified through variable importance scores. In particular, the conditional variable importance estimation procedure of [48] was used due to the inter-subject correlation structure observed between features (sample correlation matrix shown in Fig 3). To better assess predictive performance and provide more stable estimates of variable importance, repeated 5-fold cross-validation over thirty replications was used.

### Comparison of predictive performance to static connectivity and traditional estimates of dFC

Predictive performance of our approach for predicting disease class on the individual subject level was assessed using classification accuracy:

$$\begin{array}{c} \text{Accuracy}=\frac{TP+TN}{TP+FP+FN+TN}, \end{array}$$

where TP is the number of true positives; TN is the number of true negatives; FP is the number of false positives; and FN is the number of false negatives. Repeated 5-fold cross-validation was used to compare predictive accuracy using our approach to predictive accuracy using static connectivity. To investigate the additional information added by including dynamic features compared to static functional connectivity alone, we compared to predictive performance of a Random Forests classifier trained on Pearson correlation estimates of static functional connectivity (sFC-RF). For reference, performance using static functional connectivity under a logistic classifier is also shown (sFC). Lastly, we compared to predictive performance using the approach more commonly employed currently in dFC analysis, based on the mean and variance of sliding window dynamic functional connectivity, including (a) the mean of dynamic functional connectivity (SW-Mean), (b) the variance of dynamic functional connectivity (SW-Var), and (c) the mean and variance of dFC combined (SW-MeanVar). Allen and colleagues (2014) studied varying window sizes and overlap for estimating dynamic functional connectivity and found that window sizes of 30s to 2min have little effect on functional dynamics [49]. Here, a sliding window size of 80s with 50% overlap was used.

## Results

### Resting-state network differences in dynamic functional connectivity

**Fig 4:**
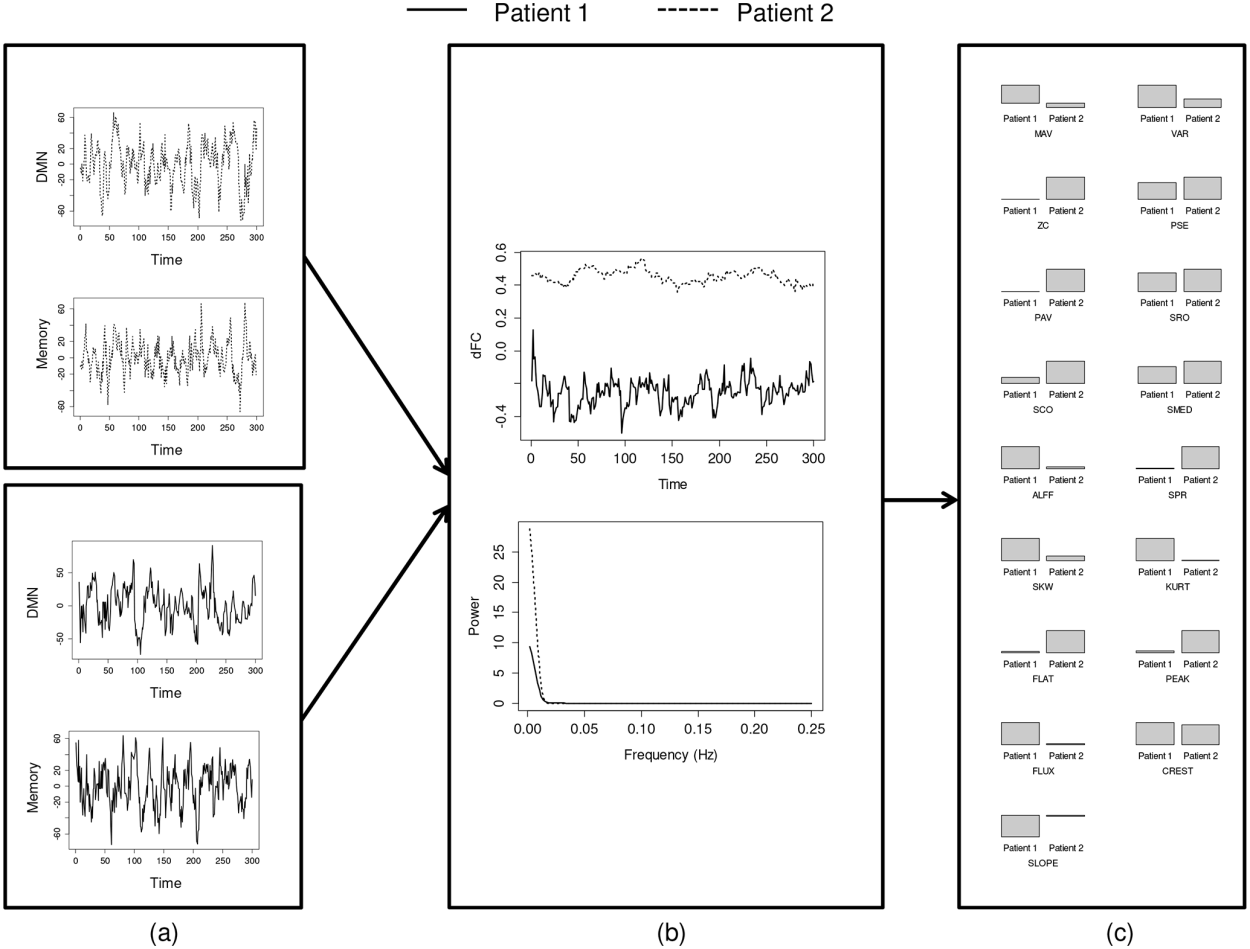
Estimated dynamic functional connectivity, power spectra, and dFC features for connectivity between the DMN and memory network for two sample patients with temporal lobe epilepsy. (a) original BOLD time-series, (b) estimated dynamic functional connectivity and dFC power spectra, and (c) estimated dFC features. *Abbreviations:* MV, Mean Value; VAR, Variance; ZC, Zero Crossing Rate; PSE, Power Spectral Entropy; PAV, Proportion of Anticorrelated Volumes; SRO, Spectral Rolloff; SCO, Spectral Centroid; SMED, Median Frequency; ALFF-dFC, Amplitude of Low Frequency Fluctuations in dFC; SPR, Spectral Spread; SKW, Spectral Skewness; KURT, Spectral Kurtosis; FLAT, Spectral Flatness; PEAK, Spectral Peak; FLUX, Spectral Flux; CREST, Spectral Crest; SLOPE, Spectral Slope.

Our approach was applied to two populations of interest in brain connectivity research, healthy controls and patients with temporal lobe epilepsy. Fig 4 shows the estimated dynamic functional connectivity, dFC power spectra, and estimated temporal/spectral dFC features for connectivity between the DMN and memory network for two sample TLE patients.

**Fig 5:**
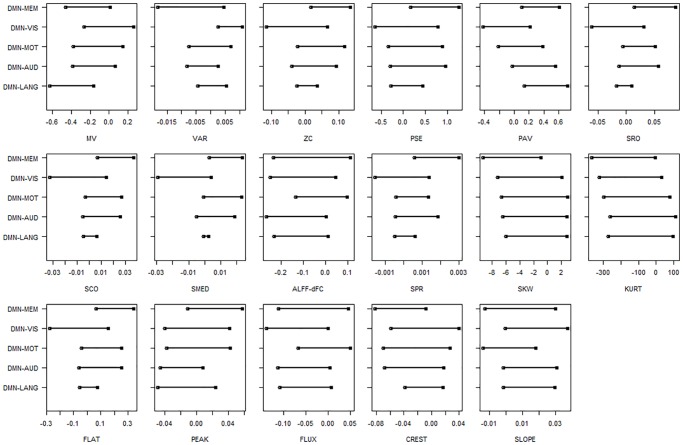
95% bias-corrected and accelerated (BCa) bootstrap confidence intervals for the difference in means (HC-TLE) for each dFC feature. Positive values indicate larger values in HC than in TLE. *Abbreviations:* HC, healthy control; TLE, temporal lobe epilepsy; MV, Mean Value; VAR, Variance; ZC, Zero Crossing Rate; PSE, Power Spectral Entropy; PAV, Proportion of Anticorrelated Volumes; SRO, Spectral Rolloff; SCO, Spectral Centroid; SMED, Median Frequency; ALFF-dFC, Amplitude of Low Frequency Fluctuations in dFC; SPR, Spectral Spread; SKW, Spectral Skewness; KURT, Spectral Kurtosis; FLAT, Spectral Flatness; PEAK, Spectral Peak; FLUX, Spectral Flux; CREST, Spectral Crest; SLOPE, Spectral Slope.

Clinical deficits in memory are often reported by patients with temporal lobe epilepsy [50–53]. The largest number of significantly altered characteristics of dFC was found for connectivity between the DMN and memory network. Connectivity between the DMN and memory network in TLE patients differed significantly from controls with respect to the zero-crossing rate (*p* = 0.008), power spectral entropy (*p* < 0.001), proportion of anticorrelated volumes (*p* < 0.001), spectral roll-off (*p* = 0.014), spectral centroid (*p* = 0.008), spectral median (*p* = 0.045), spectral spread (*p* < 0.001), spectral skewness (*p* = 0.008), spectral kurtosis (*p* = 0.03), spectral flatness (*p* < 0.001) and spectral crest (*p* < 0.001). Connectivity between the DMN and visual network was significantly different in TLE with respect to the variance of dFC (*p* < 0.001) and spectral flux (*p* = 0.045). Connectivity between the DMN and language network was significantly different for the mean value of dFC (*p* < 0.001) and proportion of anticorrelated volumes (*p* < 0.001). Estimated 95% BCa bootstrap confidence intervals for the difference in means for each proposed dFC feature are shown in Fig 5.

A number of between-network differences were also noted for both TLE and healthy controls, primarily involving the mean value and proportion of anticorrelated volumes. For more details on between-network differences, we refer the readers to the Supplementary Material.

### Prediction of disease status with proposed dFC features

**Fig 6:**
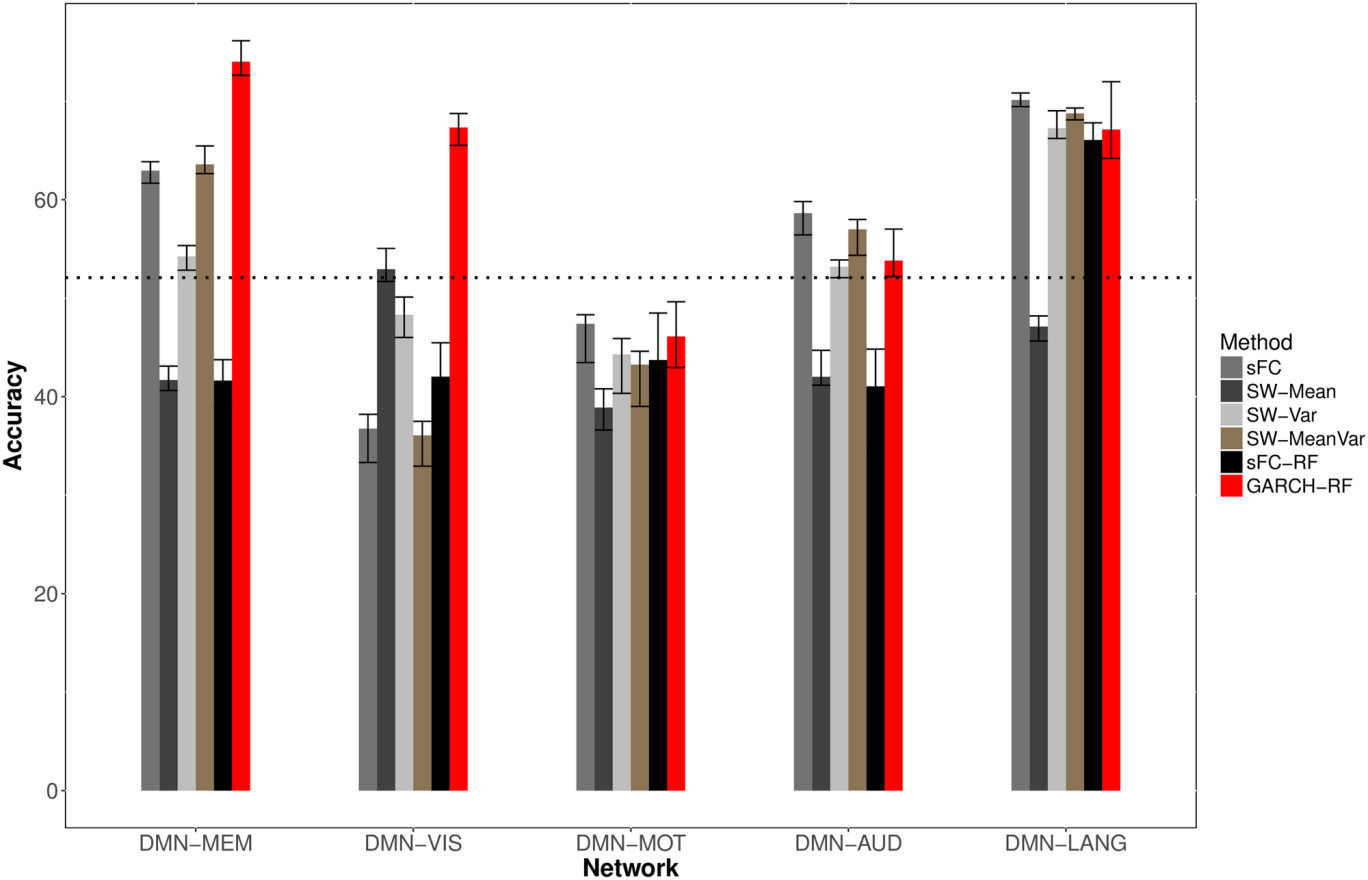
Predictive performance of dFC features. Classification accuracy (TLE vs. controls) based on connectivity between DMN and various resting-state networks is shown. The horizontal line indicates the performance of a naïve classifer. The naïve classifer provides a baseline level of performance and classifies all test samples as the most common class in the training set. Static functional connectivity (sFC; cyan), dFC mean (SW-Mean; purple), dFC variance (SW-VAR; gray), dFC mean and variance (SW-MeanVar; green), static functional connectivity with random forests (sFC-RF), and proposed dFC approach (GARCH-RF; red). Mean and 95% CI over thirty replicates are shown.

Fig 6 compares predictive performance using the proposed approach to prediction using static functional connectivity (e.g., the Pearson correlation) and prediction based on the mean and/or variance of dFC. To provide a baseline for comparison, the null rate based on a naïve classifier is also shown (horizontal line). We found that our approach provides higher predictive accuracy for the DMN/memory and DMN/visual networks compared to static functional connectivity as well as traditional methods (e.g. the first two moments of dynamic functional connectivity). Compared to static connectivity, our approach obtains 11% and 31% higher accuracies in differentiating TLE patients from healthy controls using connectivity between the DMN/memory and DMN/visual networks, respectively. For the DMN/motor, DMN/auditory, and DMN/language networks, equivalent predictive performance was observed. Connectivity between the DMN and motor networks was found to be uninformative for discriminating TLE from healthy controls, with no added improvement over the null rate based on a naïve classifier.

**Fig 7:**
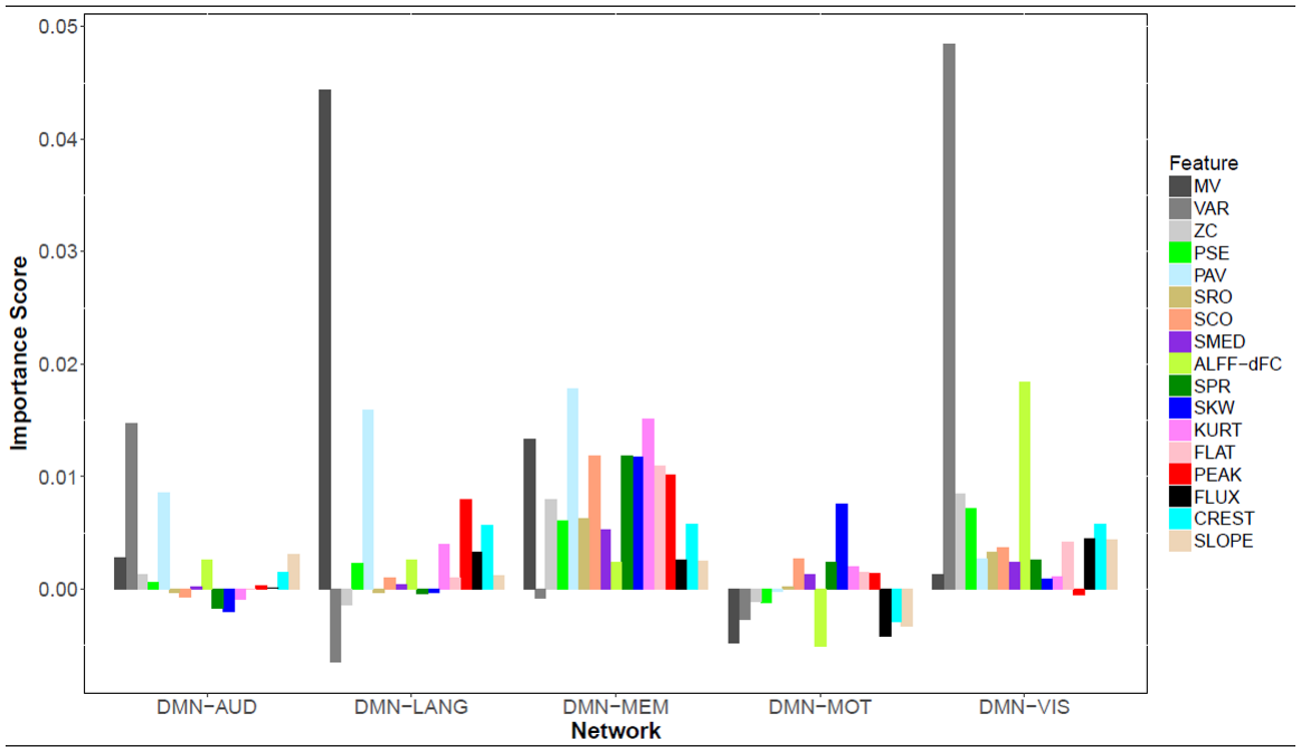
Conditional variable importance scores of dFC features. Mean of Random Forest conditional variable importance scores over 30 replicates, for dFC features between DMN and various resting-state networks. *Abbreviations:* MV, Mean Value; VAR, Variance; ZC, Zero Crossing Rate; PSE, Power Spectral Entropy; PAV, Proportion of Anticorrelated Volumes; SRO, Spectral Rolloff; SCO, Spectral Centroid; SMED, Median Frequency; ALFF-dFC, Amplitude of Low Frequency Fluctuations in dFC; SPR, Spectral Spread; SKW, Spectral Skewness; KURT, Spectral Kurtosis; FLAT, Spectral Flatness; PEAK, Spectral Peak; FLUX, Spectral Flux; CREST, Spectral Crest; SLOPE, Spectral Slope.

The conditional variable importance score of each proposed dFC feature for discriminating TLE from healthy controls is shown in Fig 7. These scores estimate the contribution of each dFC feature for subject classification based on mean decrease in accuracy. The mean value and variance of dFC, two quantities which have been considered most commonly to date in dFC investigations, were found to have relatively high levels of variable importance across most networks (black and dark gray bars). This result corroborates the importance of static connectivity and the variance of dFC in connectivity research to date. However, as shown in Fig 7, several other features of connectivity contained a significant amount of FC signal in discriminating disease groups. In particular, for the network interactions for which our method resulted in higher predictive accuracy (DMN-MEM and DMN-VIS), several features had higher variable importance than the mean or variance of dFC. In the DMN-VIS network interaction, the variance (VAR) of dFC was found to be the most important feature. However, as Fig 7 shows, the amplitude of low frequency fluctuations (ALFF-dFC) contained a larger amount of signal than the mean value (MV). For DMN-VIS interactions, the mean value, which is a measure analogous to static connectivity, was in fact identified as a poor predictor of DMN-VIS connectivity differences between TLE and HC.

With regards to DMN-MEM, the mean value (MV) of network interactions was contributed highly to group differences in connectivity, providing a potential explanation for why prior static connectivity analyses have consistently identified significant network differences between TLE and controls using Pearson correlations. However, as shown in Fig 7, the proportion of anticorrelated volumes (PAV) may provide more information than the Pearson correlation about changes in memory in TLE patients. The five most important connectivity features in discriminating TLE from healthy controls for each resting-state network are listed in Table 3.

**Table 3:** Ranked top five dFC features for discriminating TLE patients from healthy controls for various resting-state networks. (a) DMN/auditory network,(b) DMN/language network, (c)DMN/memory network, (d) DMN/motor network, (e) DMN/visual network. ALFF-dFC, amplitude of low-frequency fluctuations in dFC; CREST, spectral crest; KURT, spectral kurtosis; MV, mean value; PAV, proportion of anticorrelated volumes; PEAK, dominant frequency; PSE, power spectral entropy; SCO, spectral centroid; SKW, spectral skewness; SLOPE, spectral slope; SPR, spectral spread; VAR, variance.

Feature rank	(a) DMN-AUD	(b) DMN-LANG	(c) DMN-MEM	(d) DMN-MOT	(e) DMN-VIS
1	VAR	MV	PAV	SKW	VAR
2	PAV	PAV	KURT	SCO	ALF-dFC
3	SLOPE	PEAK	MV	SPR	ZC
4	MV	CREST	SCO	KURT	PSE
5	ALF-dFC	KURT	SPR	FLAT	CREST

## Discussion

In this work, we investigate a number of previously unexplored temporal and spectral characteristics of dynamic functional connectivity between the DMN and intrinsic connectivity networks. We show that a number of unexplored temporal and spectral dFC features exist, which are useful for understanding the dynamics of functional connectivity and may have utility in increasing the sensitivity of future connectomic analyses. Our feature engineering approach provides an interpretable way to capture the complex information present in dFC and integrate this information into subject-level prediction, which provides deeper insight into the specific aspects of dynamic functional connectivity that are altered in disease pathology. We estimate normal levels of the proposed dFC features in healthy controls and in temporal lobe epilepsy patients, which allows for insight into which particular aspects of dFC are abnormal in TLE resting-state networks. In our application of this framework to temporal lobe epilepsy, we show that there are several dFC characteristics other than the traditionally considered mean and variance which are abnormal in epilepsy. We show that inclusion of these features into machine learning may permit increased sensitivity for disease detection.

Our observation that intrinsic functional networks differ with respect to specific features of dFC suggests that the features proposed in our work may have potential clinical utility in delineating areas of cortex associated with specific tasks. This is a task which is of interest in applications such as epilepsy presurgical planning. Task-based fMRI has traditionally been used for this purpose, with newer methods utilizing seed-based cross-correlation analysis to identify areas of eloquent cortex such as the sensorimotor or language networks. Cross-correlation approaches typically rely on estimates of static connectivity, computed based on the Pearson correlation, to produce projected network maps. Although such methods have worked well for delineation of sensorimotor cortex, fMRI studies have historically reported poor positive predictive value for delineating language cortex [54, 55]. Generation of feature maps based on this expanded set of dFC features may allow for more accurate delineation of these functional networks and is a potentially exciting topic of future investigation.

Our proposed approach to dFC is novel in the respect that we investigate a large number of previously unexplored quantities in dFC between intrinsic functional networks. This is in contrast to previous studies, which have typically focused on the first- and second-order moments of dFC fluctuations. Previous work has found that static functional connectivity and the variability of dynamic fluctuations [9, 13, 17, 18, 56] elucidate pathology in neurological disorders such as temporal lobe epilepsy. Our study corroborates the reliability of the mean and variance for FC characterization, by showing that these features often capture a large amount of the signal which is useful in discriminating patient groups.

Although our results confirm the importance of the mean and variance of dFC in signal processing, our examination of variable importance scores shows that limiting dFC assessment to only these features may ignore information present in dFC fluctuations that may potentially increase the accuracy of dFC in machine learning. The physiologic implications of these features and how group differences between patients with epilepsy and healthy subjects relate to brain function is of great interest. It is thought that flexibility in cognitive processing, such as that required by higher-order association cortices, results from the ability of regions to exhibit more dynamic variability over different network configurations [42, 57]. Supporting this hypothesis, homotopic regions (corresponding regions in opposite hemispheres) have been found to have the lowest variability in connectivity, followed by regions within sensory and motor networks, with the greatest amount of variability between higher-order network nodes [58–60]. Regions with bidirectional anatomical projections generally exhibit more stable FC (e.g., lower variance), followed by those with unidirectional anatomical connectivity and those with no direct anatomical connectivity [61]. Due to the still emerging nature of dynamic functional connectivity, however, the physiologic meaning of the large majority of these features, such as why functional connectivity may fluctuate at a particular dominant frequency, is still unknown. To our knowledge, this study is the first to evaluate these features in functional connectivity data, and provides evidence that these features may contain useful information. Distinct EEG signatures have been found to correlate with fMRI dFC states [62], and further investigation using electrophysiologic data is of interest for illuminating underlying neurophysiological correlates.

Patients with temporal lobe epilepsy often report cognitive deficits with working memory and declarative memory [50–53]. Current research has found reduced static functional connectivity between the mesial temporal lobes and the posterior DMN in temporal lobe epilepsy, with worse memory performance correlating with more severe alterations in static functional connectivity [63, 64]. Consistent with previous studies, we found the mean value of dFC between the DMN and memory network to have high variable importance in discriminating TLE from controls. This supports the clinical relevance of our approach, and connects our results with previous research which have found replicable differences using static connectivity. However, groupwise comparison of TLE and controls in our study demonstrates the interesting finding that a number of other unexplored spectral aspects of connectivity between the DMN and memory network are abnormal in TLE, including decreased levels of the zero-crossing rate, spectral roll-off, spectral centroid, spectral median, spectral spread, spectral flatness, power spectral entropy, and proportion of anticorrelated volumes; and increased levels of the spectral skewness, spectral kurtosis, and spectral crest. The zero-crossing rate, spectral centroid, and spectral median characterize different aspects of the frequency at which FC fluctuates; for example, the zero-crossing rate measures the rate at which dFC changes signs, while the spectral centroid and spectral median characterize the center of the dFC power spectrum, with smaller spectral centroids/medians indicating that the power spectrum is concentrated at a lower frequency. The spectral roll-off, spectral spread, spectral flatness, power spectral entropy, spectral kurtosis, and spectral crest are different measures of the variability of different frequencies involved in dFC. Taken together, therefore, the alteration in these features suggests that the frequency at which FC between the DMN and memory networks fluctuates in TLE is abnormal, and specifically involves fluctuations at overall lower frequencies and with less variability. As discussed above, less dynamic variability around network configurations may result in decreased flexibility for higher-order cognitive processing [42, 57], and may explain the memory deficits commonly reported by patients with TLE. Additionally, we find that the levels of anticorrelation between the DMN/memory network and between the DMN/language network, estimated based on the proportion of anticorrelated volumes (PAV), are significantly lower in TLE than in healthy controls. As anticorrelation between resting-state networks is increasingly recognized to play a role in facilitating normal cognitive function [5, 11], the PAV feature proposed may provide a useful marker for measuring anticorrelation in future dFC studies. Furthermore, we show that inclusion of these additional features into machine learning allows for more accurate discrimination of disease status in TLE.

Interestingly, we found that temporal and spectral characteristics of the connectivity between the DMN and resting-state networks involving the temporal lobe, such as the memory and language networks, achieved the highest accuracy in discriminating temporal lobe epilepsy patients from healthy controls. This concurs with previous imaging research, which has most strongly implicated memory and language networks as aberrant in TLE [65], and suggests that connectivity between the DMN and memory and language networks may be more severely affected in TLE. Previous research also indicates that connectivity involving less classically involved networks, such as the visual network, may also aberrant in TLE [66], a finding which is supported here by the relatively high predictive accuracy that our approach attained using visual network connectivity. The improvement in predictive performance may be due to increased sensitivity of dynamic functional connectivity estimates to pathological inputs from other areas or cognitive processes. For example, although temporal lobe epilepsy may not directly affect the occipitally located visual network, inputs from the temporal lobe to the visual network are likely abnormal compared to that of a healthy control. Our study suggests that, while static connectivity estimates may not be sensitive to detecting abnormal inputs from other areas or cognitive processes, the added information provided by including features of dynamic connectivity may allow for improved detection of responses to these abnormal inputs.

### Limitations and future work

Although the properties of DCC-GARCH models have been studied and found to outperform current approaches for estimating dFC, particularly in data with no true correlation between time courses or with slowly varying temporal fluctuations [21], some sensitivity to noise is expected in all dynamic connectivity fMRI analysis due to the relatively limited number of sample time points [42]. Reduction of non-neural sources of noise, such as scanner drift, head motion, and non-neural physiologic signal is therefore critical in these analyses. However, even with denoising, a residual level of noise inevitably remains, and further progress in dynamic connectivity is likely to benefit highly from developments in fMRI preprocessing [42]. Additional variability is likely introduced by the use of a two-stage process to estimate dFC features, in which a single point estimate of ***R***_*it*_ is obtained and conditioned upon to estimate feature values. Two-step statistical approaches generally cause loss of information in the use of a point estimate to summarize the dynamic functional connectivity, which in turn introduces random variability in downstream estimation of dFC features. Bayesian approaches, which allow for hierarchical modeling to avoid the use of two-step processes, provide a potential solution. Methods for bootstrapping the original time-series [67] provide an alternative approach. However, while such approaches capture the variability in dFC estimates, these methods lead to computational challenges which limit the applicability of our approach to data in which large numbers of ROIs are of interest. For example, in order to accurately represent the distribution of the group means, a large number of bootstrap samples are necessary. Since for each bootstrapped time-series a DCC-GARCH model must be fit, selection of the order of the mean (ARMA) process and the covariance (GARCH) process through manual inspection of each individual bootstrapped time-series is not feasible. Automatic procedures for selecting the order of the mean and covariance process exist, but introduce their own limitations and must be weighed against potential benefit. While outside the scope of this study, these factors should be taken into consideration when interpreting results from dynamic analysis.

The framework for quantifying dynamic functional connectivity described in this paper, while examined in the context of resting-state fMRI, is broadly applicable to other electrophysiological modalities such as EEG or magnetoencephalography (MEG). The physiologic interpretation of the proposed dynamic connectivity features is intriguing and requires further investigation with EEG or MEG. Although fMRI provides the advantage of higher spatial resolution, its basis for studying neuronal activity is based on the hemodynamic response as an indirect measure of neuronal metabolism, and is potentially affected changes in blood flow induced by non-neuronal physiological processes such as cardiac and respiratory function. Future research may potentially benefit from improvements in signal by incorporating data on physiological processes. As shown in the present study, the subset of which particular temporal or spectral features are affected by different disease processes is a reflection of and may be informative about the underlying disease process, potentially analogous to the relationship of EEG slowing to pathology or PET hypometabolism to decreased neuronal activity. Applications to larger samples of healthy controls may also be useful for understanding the origin of these features.

As discussed in the *Dynamic Conditional Correlation Model* section, the evolution of dynamic connectivity is modeled in this work as a DCC-GARCH process of order one. If there is evidence of the presence of a long memory process in dynamic functional connectivity, an extension to higher orders is possible. Although no deviations from the weak stationarity assumption were observed for the current data, further investigation into the effects of such deviations on dynamic connectivity estimates might be of interest. Future work may also wish to explore a stochastic evolution of the covariance matrix rather than the deterministic assumption inherent in all GARCH type models. Although preliminary analyses comparing spectra between initial and later volumes did not suggest evolution of spectral properties over time, further extensions to allow for changing spectral properties within the scan [68] are possible within this context.

Future work may also benefit from investigation of connectivity involving higher-frequency fMRI BOLD fluctuations. In this work, fMRI volumes were collected using a TR of 2000 ms, a common sampling rate in resting-state fMRI studies. Consequently, dynamics in this study reflect connectivity between BOLD fluctuations at a maximum frequency of 0.25 Hz. Some recent research suggests that common resting-state networks emerge at frequencies even up to 1.4 Hz, and that higher frequencies may also contain meaningful information about resting-state networks [34]. The hemodynamic response that produces BOLD signal has a long time decay over seconds, rendering fMRI insensitive to shorter duration (higher frequency) activity. Additional work is needed to understand the additional information about dynamic connectivity that may be gained by sampling at higher frequencies.

Additional limitations of the approach utilized here suggest avenues for future work. In the present work, we focus on dynamic connectivity between the DMN and several commonly examined resting-state networks in resting-state fMRI. However, it is important to note that the proposed framework is applicable to the connectivity between any choice of regions, including connectivity between voxels, regions, or other resting-state networks. Further insight may be obtained by investigating the properties of dynamic connectivity under networks extracted through other commonly employed methods. Although seed-based correlations and ICA have been found to yield similar networks [35], results are similar but not identical. There are various advantages and disadvantages to each approach, including the advantage of spatially orthogonal components in ICA, a constraint which is not enforced in seed-based analysis, but the disadvantage of the need to specify the number of components in ICA. Comparison to an ICA-based approach for network extraction would be useful and is of interest in future replicability studies.

## Supporting information

S1 Table Simulation study. 95% confidence intervals (CI) for estimated dFC features.
(PDF)
