# Supplementary material for: Temporal and spectral characteristics of dynamic functional connectivity between resting-state networks reveal information beyond static connectivity
Source: PLoS One. 2018 Jan 10;13(1):e0190220. doi: 10.1371/journal.pone.0190220 (PMC5761874; doi:10.1371/journal.pone.0190220)
Supplement: S1 Table — 95% confidence intervals (CI) for estimated dFC features. (PDF) [file pone.0190220.s001.pdf]

# Supplementary Material for Temporal and spectral characteristics of dynamic functional connectivity between resting-state networks reveal information beyond static connectivity

Sharon Chiang[1,2*◑], Emilian R. Vankov[1,3◑], Hsiang J. Yeh[4¤], Michele Guindani[5], Marina Vannucci[1], Zulfi Haneef[6,7], John M. Stern[4]

**1** Department of Statistics, Rice University, Houston, Texas, USA
**2** Baylor College of Medicine, School of Medicine, Houston, Texas, USA
**3** Baker Institute for Public Policy, Rice University, Houston, Texas, USA
**4** Department of Neurology, University of California at Los Angeles, Los Angeles, California, USA
**5** Department of Statistics, Uniersity of California at Irvine, Irvine, California, USA
**6** Department of Neurology, Baylor College of Medicine, Houston, Texas, USA
**7** Neurology Care Line, Michael E. DeBakey VA Medical Center, Houston, Texas, USA

◑These authors contributed equally to this work.
* schiang@bcm.edu (SC)

## Simulation study: confidence intervals for dynamic functional connectivity features

A simulation study was conducted to compare true values of the features when the underlying correlation is fixed to a known quantity, to 95% confidence intervals for the estimated features using the DCC-GARCH procedure. Simulated true values of temporal and spectral dFC features described in Sections *Temporal dFC features* and *Spectral dFC features* were first calculated by simulating values of $\boldsymbol{R}_t$ for each subject using the GARCH model described in Section *Dynamic Conditional Correlation Model*. The mean process was specified as an ARMA(2,2). This allowed for the simulation of "true" values of temporal and spectral dFC features for each subject. In order to evaluate the ability for our estimation procedure to capture the true dFC feature values under model misspecification, 100 fMRI time-series realizations with dynamic functional connectivity given by the "true" $\boldsymbol{R}_{it}$ were calculated. A misspecified ARMA(1,1) model was then fit to all fMRI time-series. Table S1 shows the 95% confidence intervals for three sample patients. We observe that overall our estimated features capture the true values of the underlying temporal and spectral properties of dFC. Confidence intervals were wider for some features, such as KURT or PSE, than others, potentially reflecting larger contributions of the estimation procedure.

## Between-network differences in dynamic functional connectivity features among healthy controls and patients with TLE

Significant between-network differences among temporal lobe epilepsy patients were identified at the $\alpha = 0.05$ level based on the bootstrap distribution for the difference in

sample means after multiple testing correction [1]. It was found that (1) $\mathrm{MV}_{DMN/motor}$ was significantly different than $\mathrm{MV}_{DMN/memory}(p < 0.001)$ or $\mathrm{MV}_{DMN/language}(p < 0.001)$; (2) $\mathrm{PAV}_{DMN/motor}$ was significantly different than $\mathrm{PAV}_{DMN/memory}(p < 0.001)$, $\mathrm{PAV}_{DMN/auditory}(p = 0.048)$, or $\mathrm{PAV}_{DMN/language}(p < 0.001)$; and (3) $\mathrm{PAV}_{DMN/visual}$ was significantly different than $\mathrm{PAV}_{DMN/memory}(p < 0.001)$, or $\mathrm{PAV}_{DMN/language}(p < 0.001)$ No significant between-network differences were identified at the 0.05 level among healthy controls.

## References

1. Benjamini Y, Hochberg Y. Controlling the false discovery rate: a practical and powerful approach to multiple testing. Journal of the royal statistical society Series B (Methodological). 1995; p. 289–300.

**Table S1. Simulation study: 95% confidence intervals (CI) for estimated dFC features.**

| | Subject 1 | | Subject 2 | | Subject 3 | |
|---|---|---|---|---|---|---|
| | True Value | 95% CI | True Value | 95% CI | True Value | 95% CI |
| ALFF-dFC | 0.02 | $(0.01, 0.06)$ | 0.02 | $(0.004, 0.05)$ | 0.03 | $(0.01, 0.05)$ |
| CREST | 0.11 | $(0.02, 0.23)$ | 0.18 | $(0.06, 0.56)$ | 0.05 | $(0.01, 0.68)$ |
| FLAT | 0.66 | $(0.46, 0.83)$ | 0.69 | $(0.23, 0.8)$ | 0.72 | $(0.18, 0.86)$ |
| FLUX | 0.002 | $(0.001, 0.004)$ | 0.004 | $(0.002, 0.005)$ | 0.002 | $(0.001, 0.003)$ |
| KURT | $-0.61$ | $(-1.21, 0.82)$ | $-1.18$ | $(-1.27, 5.8)$ | $-0.76$ | $(-1.21, 24)$ |
| MV | $-0.14$ | $(-0.23, 0.07)$ | $-0.3$ | $(-0.41, -0.17)$ | 0.02 | $(-0.13, 0.15)$ |
| PAV | 0.94 | $(0.34, 0.99)$ | 0.98 | $(0.86, 1)$ | 0.42 | $(0, 0.98)$ |
| PEAK | 0.01 | $(0.001, 0.15)$ | 0.09 | $(0.01, 0.17)$ | 0.003 | $(0.001, 0.14)$ |
| PSE | 1.56 | $(0.52, 4.31)$ | 0.63 | $(0.03, 2.6)$ | 3.7 | $(0.2, 4.6)$ |
| SCO | 0.07 | $(0.05, 0.11)$ | 0.09 | $(0.02, 0.11)$ | 0.08 | $(0.01, 0.12)$ |
| SLOPE | $-4e^{-4}$ | $(-6e^{-4}, -3.4e^{-5})$ | $-2e^{-4}$ | $(-5.6e^{-4}, -1.3e^{-4})$ | $-2.4e^{-4}$ | $(-5.5e^{-4}, -1.2e^{-4})$ |
| SMED | 0.05 | $(0.02, 0.11)$ | 0.08 | $(0.01, 0.1)$ | 0.06 | $(0.003, 0.12)$ |
| SKW | 0.8 | $(0.1, 1.41)$ | 0.39 | $(0.16, 2.54)$ | 0.68 | $(0.05, 4.3)$ |
| SPR | 0.0051 | $(0.0043, 0.00583)$ | 0.006 | $(0.002, 0.007)$ | 0.005 | $(0.002, 0.006)$ |
| SRO | 0.17 | $(0.13, 0.2)$ | 0.2 | $(0.05, 0.21)$ | 0.18 | $(0.04, 0.21)$ |
| VAR | 0.01 | $(0.001, 0.06)$ | 0.01 | $(0.001, 0.04)$ | 0.02 | $(5.4e^{-6}, 0.05)$ |
| ZC | 0.07 | $(0.003, 0.34)$ | 0.02 | $(0, 0.18)$ | 0.23 | $(0, 0.47)$ |
